# Supplementary material for: Interlayer epitaxy of wafer-scale high-quality uniform AB-stacked bilayer graphene films on liquid Pt3Si/solid Pt
Source: Nat Commun. 2019 Jun 26;10:2809. doi: 10.1038/s41467-019-10691-2 (PMC6594936; doi:10.1038/s41467-019-10691-2)
Supplement: Supplementary file 1 — Supplementary Information [file 41467_2019_10691_MOESM1_ESM.pdf]

Supplementary Information for

Interlayer epitaxy of wafer-scale high-quality uniform AB-stacked bilayer graphene films on liquid Pt_3Si/solid Pt

Wei *et al*

Supplementary Table 1. Comparison of the structure and electrical properties of bilayer graphene (BLG) synthesized by chemical vapor deposition (CVD) and mechanical exfoliation

Methods	Substrate	Coverage ratio (%)	Ratio of AB stacking (%)	Grain size (μm)	I_{2D}/I_G	*2D* FWHM (cm^{-1})	Mobility ($cm^2\ V^{-1}\ s^{-1}$)	Reference
CVD	**Pt_3Si (L)/Pt (S)**	**100**	**100**	**~1000**	**0.51-0.83**	**52.4-57.1**	**~2100 (RT, SiO_2^{*}, $Al_2O_3^{\#}$)**	**This work**
	Cu (S)				2-2.8	42-63	580 (RT, SiO_2^{*}, $Al_2O_3^{\#}$)	*Nano Lett.* 10, 4702-4707, 2010.
		67		<50†			350-550 (RT, SiO_2^{*}, $Al_2O_3^{\#}$)	*Nano Lett.* 11, 1106-1110, 2011.
		99	90	~40†	0.83-1.46	47.5-62	1400-3000 (RT, SiO_2^{*}, $Al_2O_3^{\#}$)	*ACS Nano* 6, 8241-8249, 2012.
		~60†		300	~0.8	~58		*Nat. Commun* 4, 2096, 2013.
		~88		<40†	0.7-1.3	45-60		*ACS Nano* 8, 11631-11638, 2014.
		~75†	80	500			20000 (300 K, BN^{*}, $BN^{\#}$)	*Nat. Nanotechnol* 11, 426-431, 2016.
				<30†	0.7-1.1	40-50	930-2200 (RT, SiO_2^{*}, Ionic liquid$^{\#}$)	*Adv. Funct. Mater.* 27, 1605927, 2017.
		77 ±3.6	93±3	<10†			1100 (300 K, SiO_2^{*})	*Chem. Mater.* 30, 7852-7859, 2018.
	Ni-Cu Alloy (S)	90	87†	<50†	0.3-0.6			*Adv. Funct. Mater.* 25, 3666-75, 2015.
		90	90	~300	0.9-1.4	53-62		*Small* 12, 2009-2013, 2016.
		92	48	20†		~50	2700 ± 400 (RT, SiO_2^{*}, $HfO_2^{\#}$)	*ACS Nano* 12, 2275-2282, 2018.
ME							1000 (RT, SiO_2^{*}, $Al_2O_3^{\#}$)	*Nature* 459, 820-823, 2009.
							1000 (4.2 K, $SiO_2^{*\#}$)	*Nat. Mater* 7, 151-157, 2008.
							1700-3000 (RT, SiO_2^{*})	*Appl Phys Express* 2, 3, 2009.
							2700 (4.2 K, $SiO_2^{*\#}$)	*Nat. Nanotechnol* 4, 383-388, 2009.

ME: Mechanical exfoliation; L: Liquid; S: Solid; RT: Room temperature; *: Bottom gate; $^{\#}$: Top gate; †: Estimated based on the Figures in the corresponding references.

Supplementary Figures

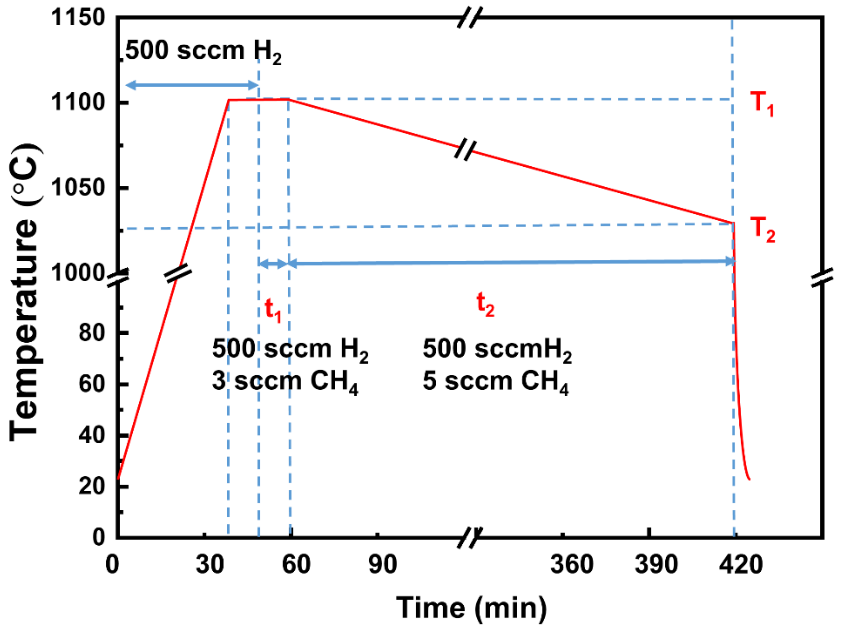

Supplementary Figure 1. A typical CVD process for the interlayer epitaxy of AB-BLG film using the core-shell structured liquid Pt_3Si/solid Pt substrate. T_1 =1100 ºC; T_2 = 1025 ºC; t_1 = 10 min; t_2 = 360 min.

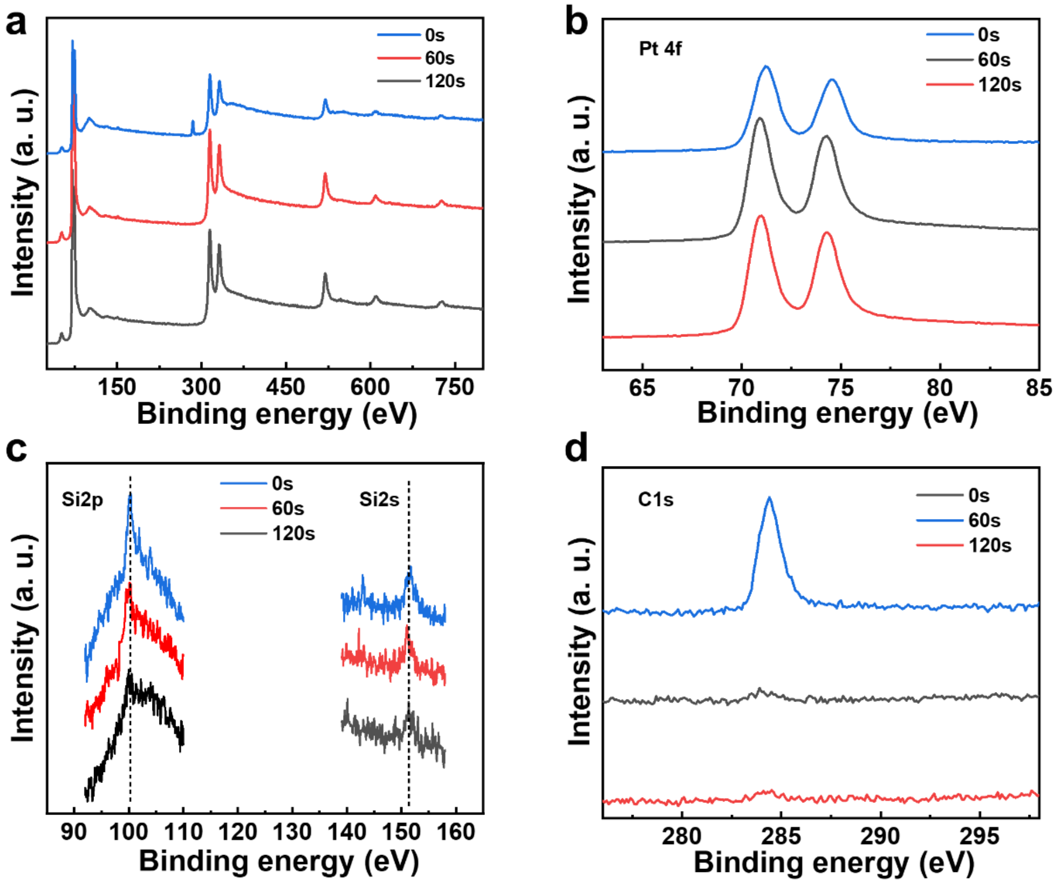

Supplementary Figure 2. XPS characterization on the Pt_3Si/Pt substrate after monolayer graphene (MLG) growth. a, XPS spectra of the Pt_3Si/Pt substrate etched by Ar^+ for 0 s, 60 s and 120 s. The etching rate is ~0.1 nm s^{-1}. **b-d,** The corresponding Pt4f (**b**), Si2p and Si2s (**c**), and C1s (**d**) spectra. The Pt_3Si/Pt substrate was quickly pulled out of the high-temperature zone after MLG was grown on it at 1100 ºC in 10 min. Such operation ensures a rapid quenching of the reactions and allows the carbon distribution in the substrate during growth to be captured. Because of the presence of MLG, strong C1s peak was observed in the substrate without Ar^+ etching. After 60 and 120 s etching, the Pt_3Si is exposed and shows a low C1s peak, indicating that the liquid Pt_3Si has a low carbon solubility.

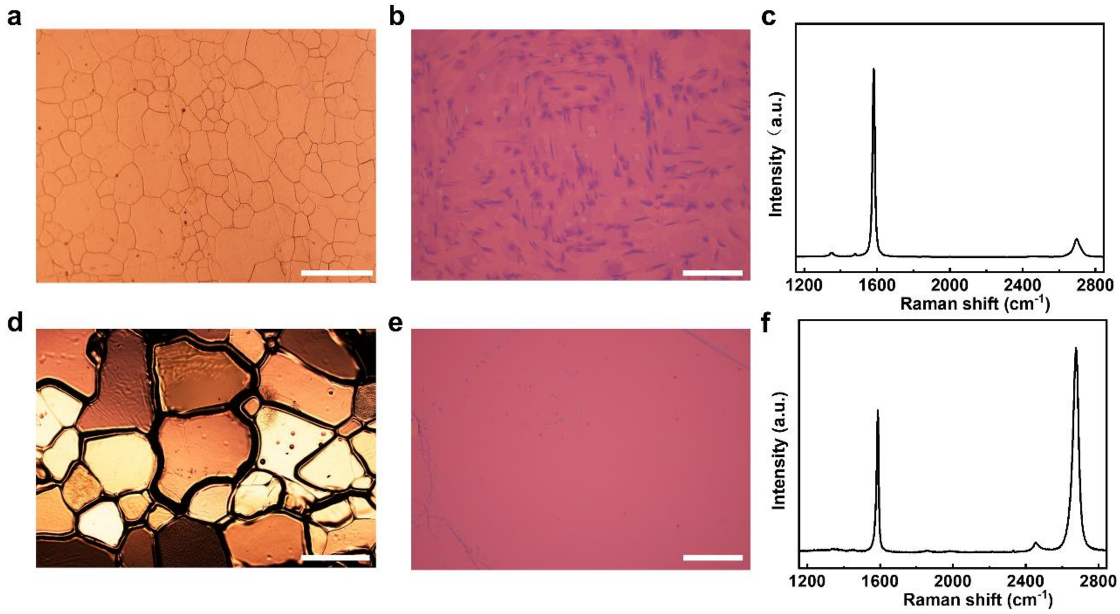

Supplementary Figure 3. Graphene grown on Pt and Pt_3Si substrates. a-c, Optical images of Pt substrate (**a**), graphene grown on Pt substrate and then transferred on SiO_2/Si substrate (**b**), and Raman spectrum (**c**) taken from the graphene in (**b**). **d-f,** Optical images of Pt_3Si substrate (**d**), graphene grown on Pt_3Si and then transferred on SiO_2/Si (**e**), and Raman spectrum (**f**) taken from the graphene in (**e**). The growth processes for these two cases were the same as those used for the growth of AB-BLG film. Optical images and Raman spectra show that only non-uniform multilayers and uniform MLG film were synthesized on Pt and Pt_3Si substrate, respectively. Scale bars: **a,d**, 500 μm; **b,e**, 100 μm.

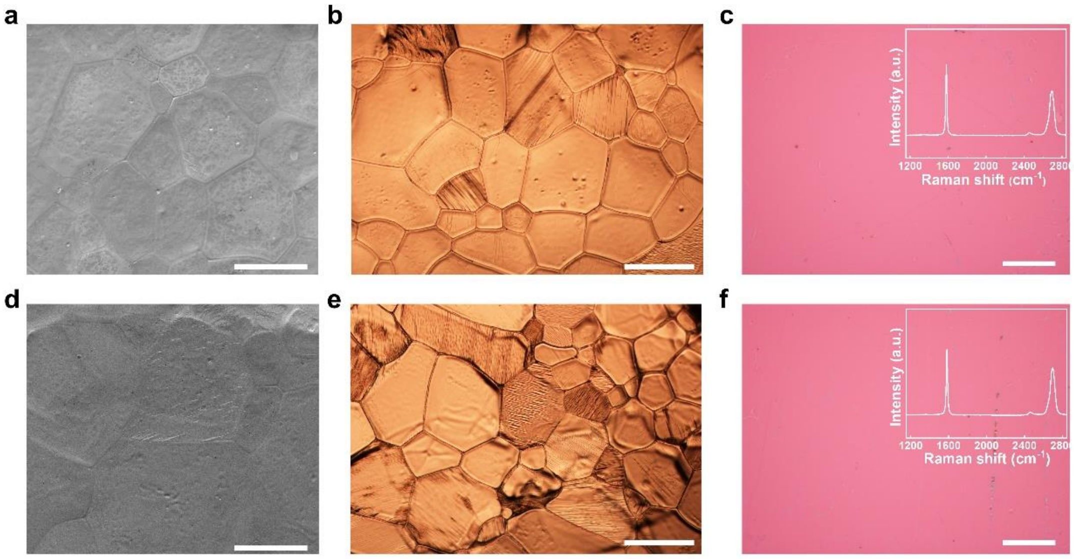

Supplementary Figure 4. Graphene growth on the reusable Pt_3Si/Pt substrate. a,b SEM (**a**) and optical (**b**) images of Pt_3Si/Pt substrate grown with AB-BLG film. **c**, Optical image of the AB-GLG film transferred on SiO_2/Si substrate by electrochemical bubbling. **d,e,** SEM (**d**) and optical (**e**) images of the Pt_3Si/Pt substrate in (**a**) and (**b**) after electrochemical bubbling transfer. **f**, Optical image of the AB-BLG film grown on the reused Pt_3Si/Pt substrate in (**d**) and (**e**) and then transferred on SiO_2/Si. Insets in (**c**) and (**f**) are the corresponding typical Raman spectra of the grown graphene films, both of which show typical features of AB-BLG. Scale bars: **a,b,d,e**, 500 μm; **c,f**, 100 μm.

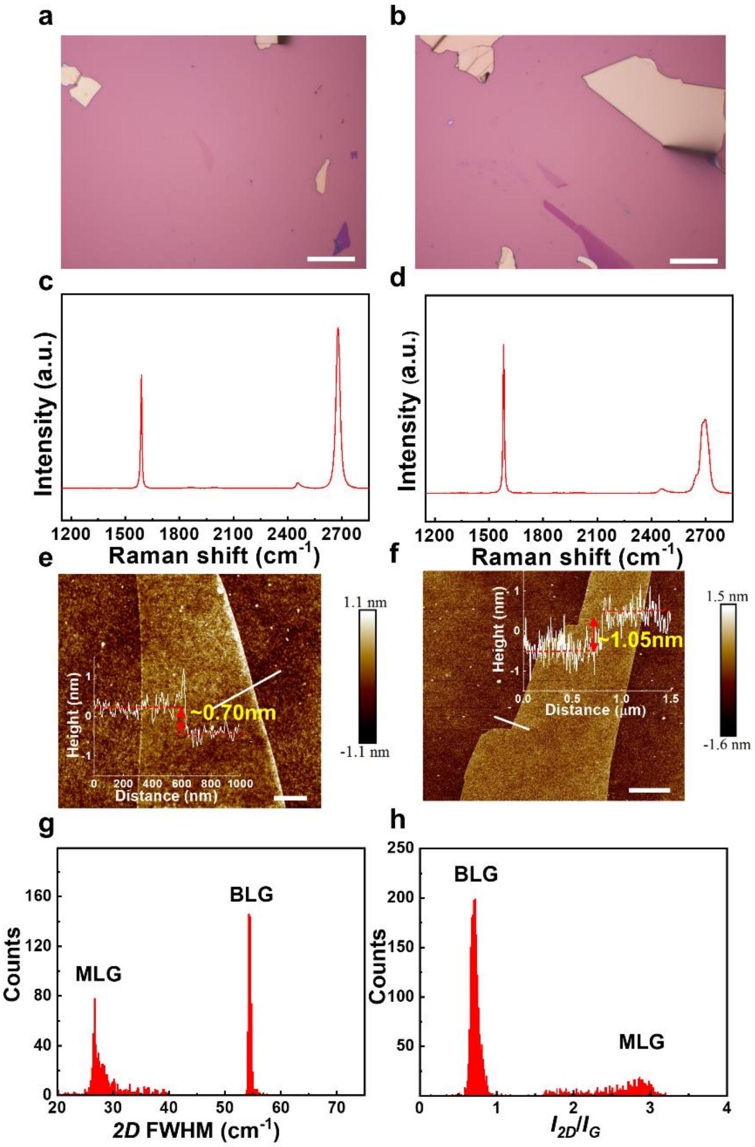

Supplementary Figure 5. Characterization of the mechanically exfoliated MLG and AB-BLG. a,b, Optical images of MLG (**a**) and BLG (**b**) on SiO_2/Si. **c,d,** Typical Raman spectra of MLG (**c**) and BLG (**d**). **e,f,** AFM images of MLG (**e**) and BLG (**f**). The insets show the height profiles along the white lines. **g,h,** Statistical histograms of *2D* peak FWHM (**g**) and I_{2D}/I_G (**h**) of the exfoliated MLG and BLG. The *2D* peak FWHM is in the range of 26.0 – 30.4 cm^{-1} and 53.7 – 56.5 cm^{-1} for MLG and BLG, respectively. The I_{2D}/ I_G is in the range of 2.5 – 3.2 and 0.5 – 0.9 for MLG and BLG, respectively. Scale bars: **a,b**, 20 μm; **e**, 500 nm; **f**, 1μm.

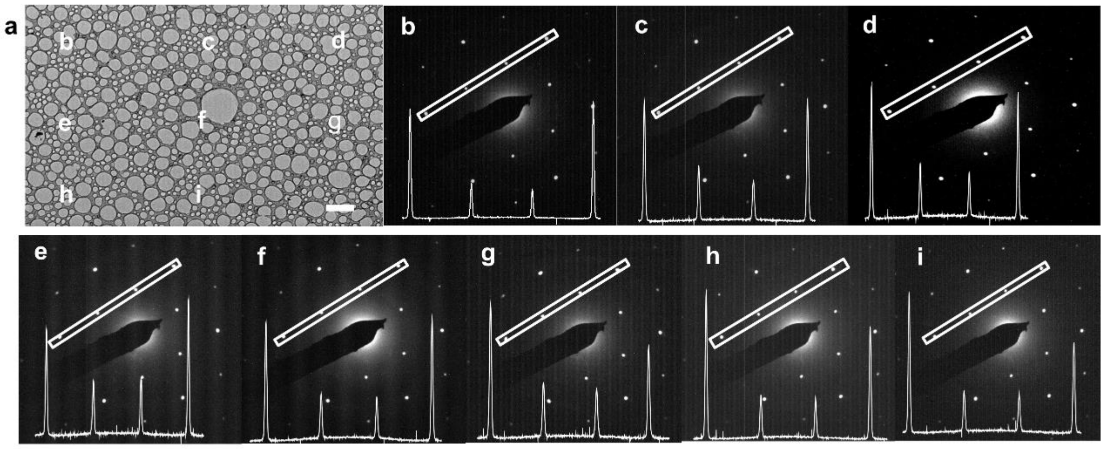

Supplementary Figure 6. SAED characterization of the CVD-grown AB-BLG film by interlayer epitaxy. a, TEM image of a CVD-grown film that was transferred onto a TEM grid. **b-i,** SAED patterns taken from 8 representative regions indicated in (**a**), showing these regions are all AB-BLG with the same lattice orientation. Scale bar: **a**, 5 μm.

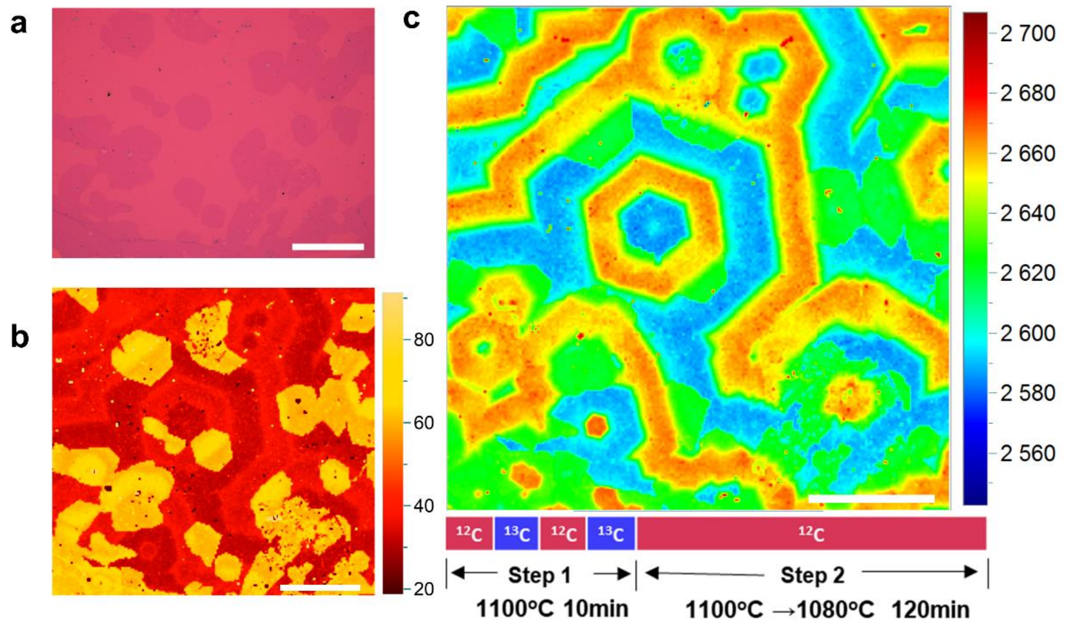

Supplementary Figure 7. Raman characterization on the isotopically labeled AB-BLG. a-c, Optical image (**a**), and the corresponding Raman maps of *2D*-peak FWHM (**b**) and position (**c**) of the CVD-grown AB-BLG transferred on SiO_2/Si substrate. $^{12}CH_4$ and $^{13}CH_4$ were alternated for periods of 2.5 min in the first step to form MLG film (in total 10 min), followed by using only $^{12}CH_4$ in the slow-cooling step. To clearly show the growth process of the second layer, we synthesized MLG film covered with isolated second layer domains as shown here by intentionally reducing the cooling time. The isotopic rings of the first layer indicate that the grain size of the first layer can reach millimeter size. Therefore, the grain size of the finally formed continuous AB-BLG film (Figure 1f) with extending the cooling time is on the order of millimeter due to the interlayer epitaxial growth mechanism. Scale bars: **a-c**, 500 μm.

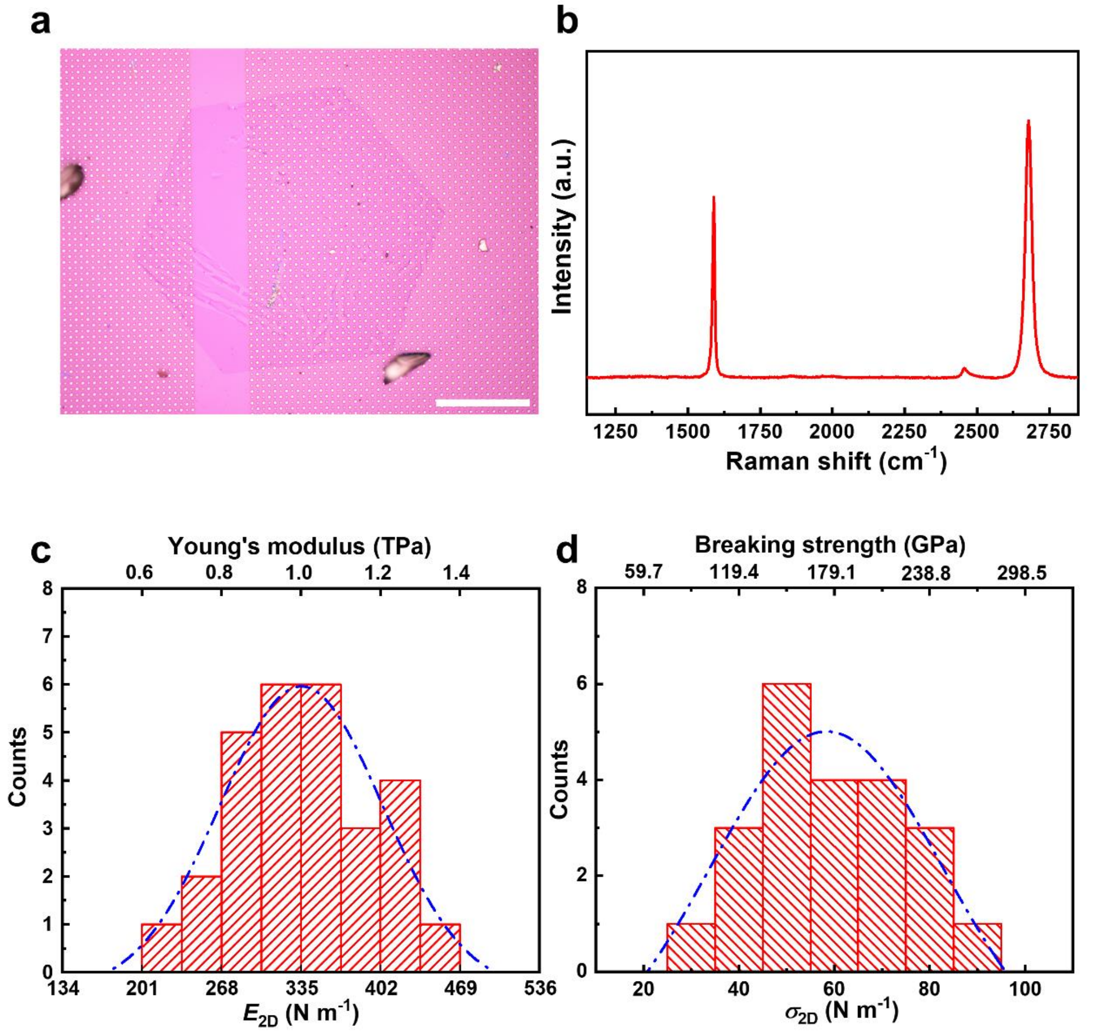

Supplementary Figure 8. Mechanical properties of the CVD-grown single-crystal MLG domains. a,b, Optical image (**a**) and Raman spectrum (**b**) of the CVD-grown single-crystal MLG transferred onto a SiO_2/Si substrate with an array of holes with 1-μm diameter. **c,d,** Histograms of elastic stiffness (**c**) and breaking strength (**d**). Dashed lines represent Gaussian fits to the data. The average 2D Young's modulus and breaking strength is ~334.02 N $m^{-1}$and ~55.31 N m^{-1}, respectively. Scale bar: **a**, 50 μm.

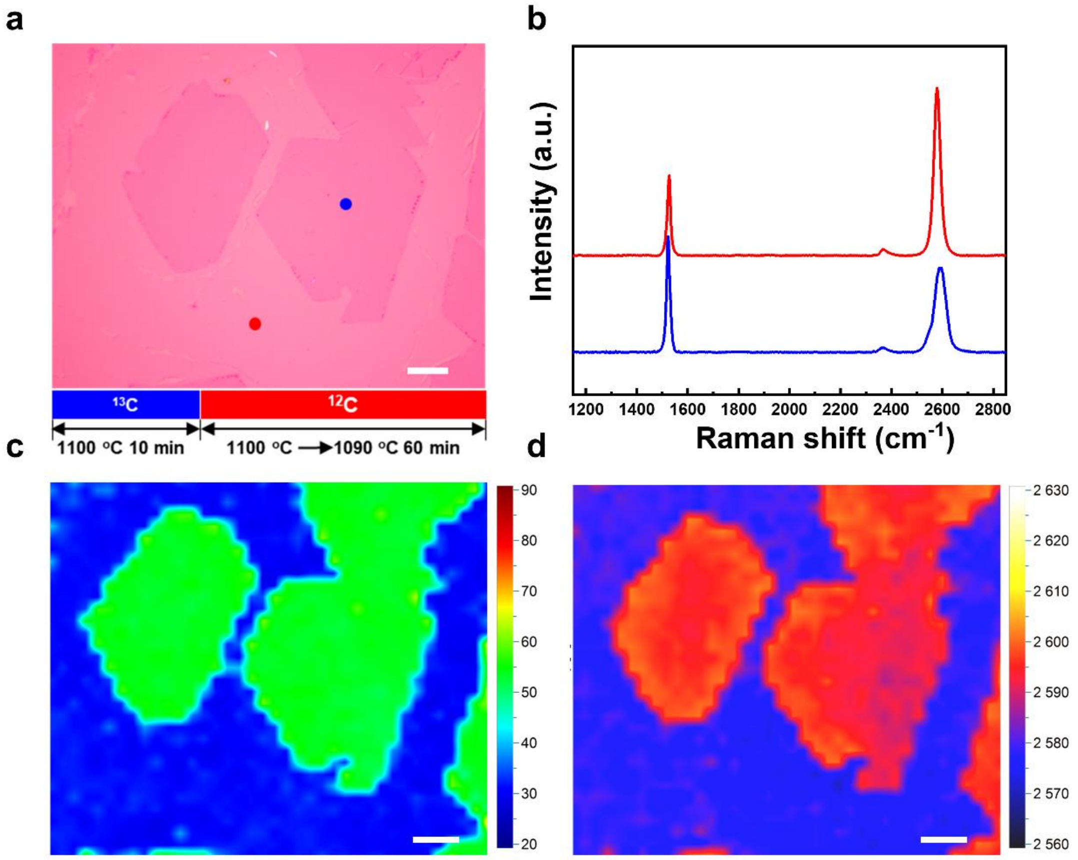

Supplementary Figure 9. Isotope experiment of growing AB-BLG film on the Pt_3Si/Pt substrate using one single isotope in each step. a-d, Optical image (**a**), Raman spectra (**b**) taken from the two regions marked in (**a**), and Raman maps of *2D*-peak FWHM (52.1 – 56.3 cm^{-1}) (**c**) and position (2590 – 2600 cm^{-1}) (**d**) of the CVD-grown AB-BLG transferred on SiO_2/Si substrate. $^{13}CH_4$ was only supplied in the first step (in total 10 min), followed by using only $^{12}CH_4$ in the second step for 60 min. Scale bars: **a,c,d**, 20 µm.

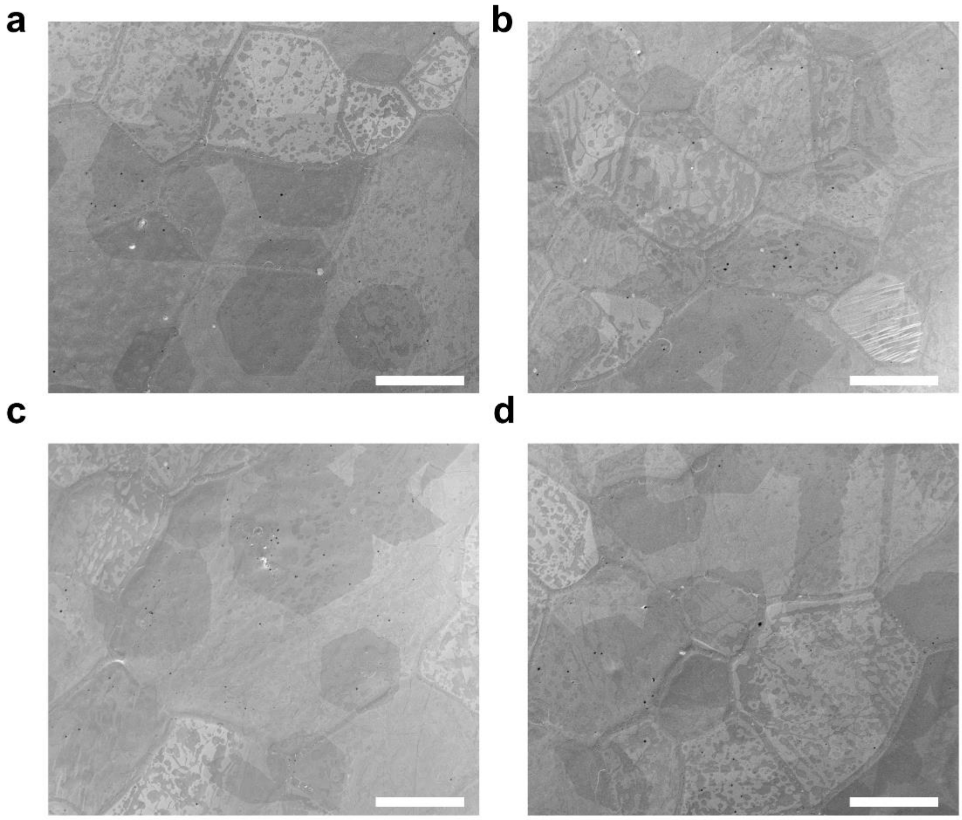

Supplementary Figure 10. SEM images of BLG domains grown on different regions of a Pt_3Si/Pt substrate. T_1 =1100 ºC; T_2 = 1075 ºC; t_1 = 10 min; t_2 = 120 min. Scale bars: **a-d**, 200 μm.

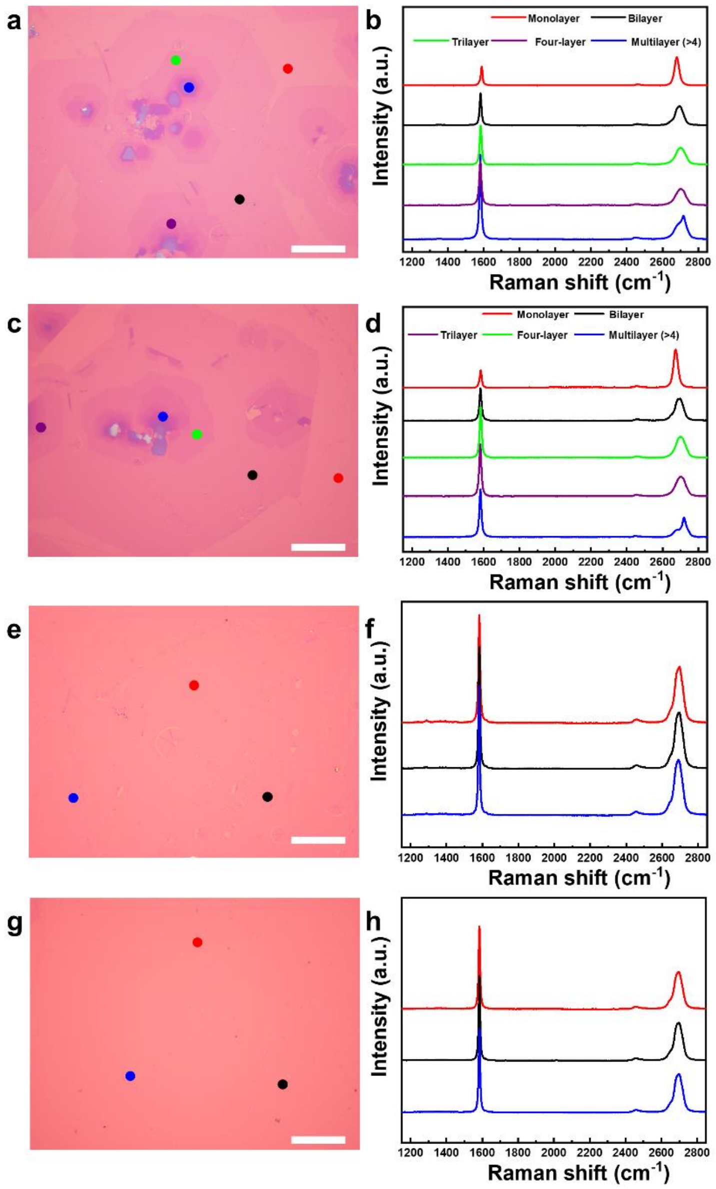

Supplementary Figure 11. Graphene growth with different cooling rates in the second step. a,c,e,g, Optical images of graphene transferred on SiO_2/Si substrates. The cooling rates are 32.5 ºC h^{-1} (**a**), 18.75 ºC h^{-1} (**c**), 12.5 ºC h^{-1} (**e**), and 9.375 ºC h^{-1} (**g**) in the cooling step, respectively. T_1 =1100 ºC; T_2 = 1025 ºC; t_1 = 10 min. **b,d,f,h**, Raman spectra taken for the regions marked in (**a**), (**c**), (**e**) and (**g**), respectively. All the bilayer regions obtained in (**a**), (**c**), (**e**) and (**g**) are AB-stacked (*2D* peak FWHM: 52.85 – 56.23 cm^{-1}), trilayer and four-layer regions in (**a**) and (**b**) are ABA-stacked (*2D* peak FWHM: 61.65 – 64.59 cm^{-1}) and ABAB-stacked (*2D* peak FWHM: 64.59 – 67.53 cm^{-1}), according to Ref 1. Scale bars: **a,c,e,g**, 100 µm.

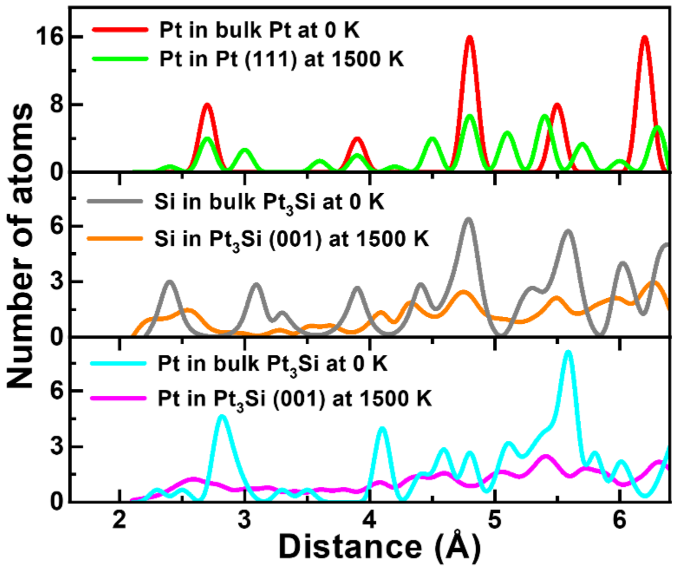

Supplementary Figure 12. The radical distribution function (RDF) plots of bulk Pt at 0 K, Pt (111) at 1500 K, bulk Pt_3Si at 0 K and Pt_3Si (001) at 1500 K. As we can see, the RDF plot of Pt atom in Pt (111) at 1500 K shows obvious peaks and well reproduces the RDF feature of Pt atom in bulk Pt at 0 K, implying that Pt (111) remains a solid state at 1500 K. This can be easily understood since 1500 K is much lower than the melting point (around 2040 K) of bulk Pt. As for Pt_3Si (001) at 1500 K, the RDF plots for both the Pt and Si atoms are very smooth with no obvious peak and show a typical RDF feature of liquid, compared with those of bulk Pt_3Si at 0 K. This is due to the low melting point (around 1151.5 K) of bulk Pt_3Si. Therefore, if we heat the Pt_3Si substrate at 1500 K, both the Pt and Si atoms should take disordered distribution. Interestingly, we have observed that one C atom can detach from the graphene nucleus and dissolve into the Pt substrate in the case of graphene (Gr)/Pt (111) at 1500 K (see Figure 4e in the main text), while the C54 graphene nucleus remains its integrity on Gr/Pt_3Si (001) at 1500 K. This might come from the different carbon solubility in Pt and Pt_3Si.

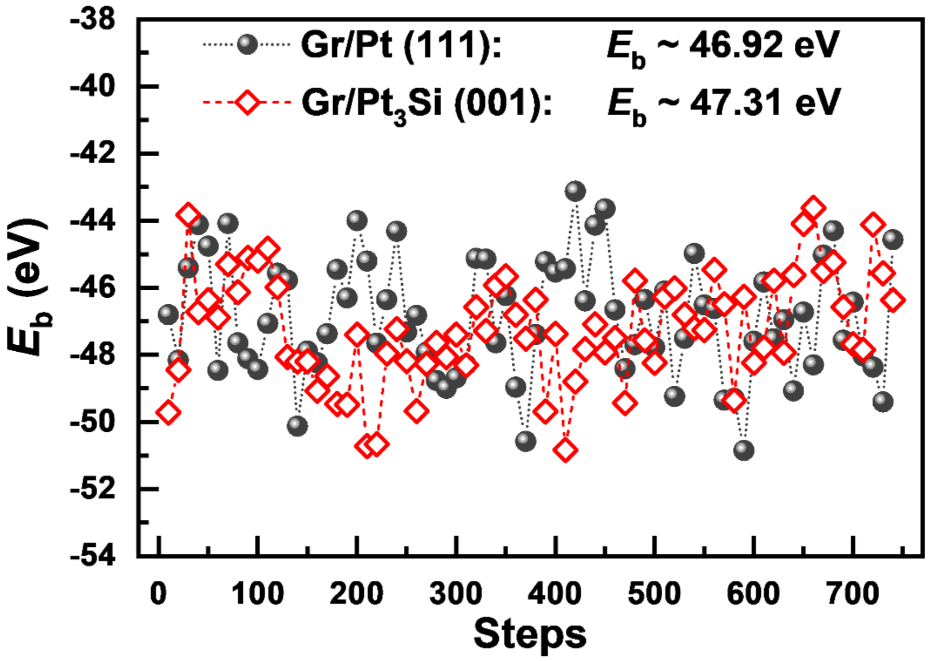

Supplementary Figure 13. The calculated binding energy (E_b) for graphene nucleus on Pt (111) and Pt_3Si (L) surfaces for selected 74 structures from the last 740 ab initio molecular dynamics (MD) simulation steps at 1500 K. For both Gr/Pt (111) and Gr/Pt_3Si (L), we took one out of every ten structures from the last 740 ab initio MD simulation steps to evaluate the E_b for graphene nucleus on Pt (111) and Pt_3Si (L) surfaces. Note that the E_b between graphene nucleus and Pt (111) is very similar to that between graphene nucleus and Pt_3Si (L). The averaged E_b for selected 74 structures are 46.92 and 47.31 eV for Gr/Pt (111) and Gr/Pt_3Si (L), respectively. This implies that the difference in E_b between these two cases is negligible, thus, we can exclude the role of binding strength in the growth behavior of the bottom graphene layer on these two different substrates.

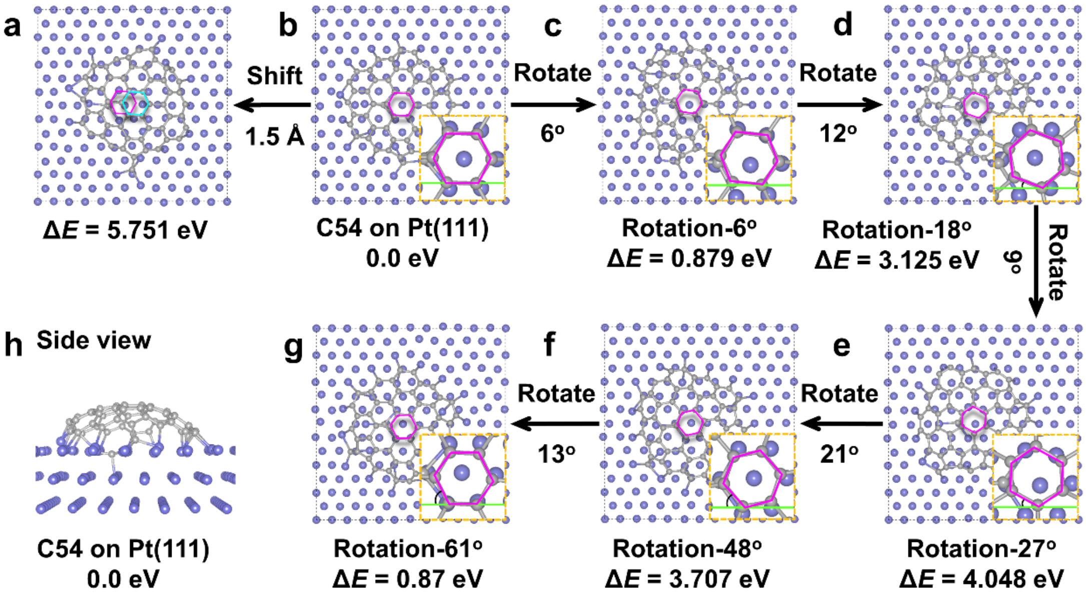

Supplementary Figure 14. The schematic diagrams of the atomic structures of graphene nucleus (C54) with different orientations and locations on Pt (111) surface. **a**, The schematic diagram of the atomic structure of C54 on Pt (111) surface after shifting 1.5 Å from (**b**). **b**, The schematic diagram of the initial atomic structure of C54 without movement on Pt (111) surface. **c-g**, The schematic diagrams of the atomic structures of C54 on Pt (111) surface after rotating 6° (**c**), 18° (**d**), 27° (**e**), 48° (**f**) and 61° (**g**) from (**b**). **h**, The side-view schematic diagram of the atomic structure of C54 on Pt (111) surface. The relative energies of all structures were calculated taking the structure with the lowest total energy as the reference, and shown below each corresponding schematic diagram.

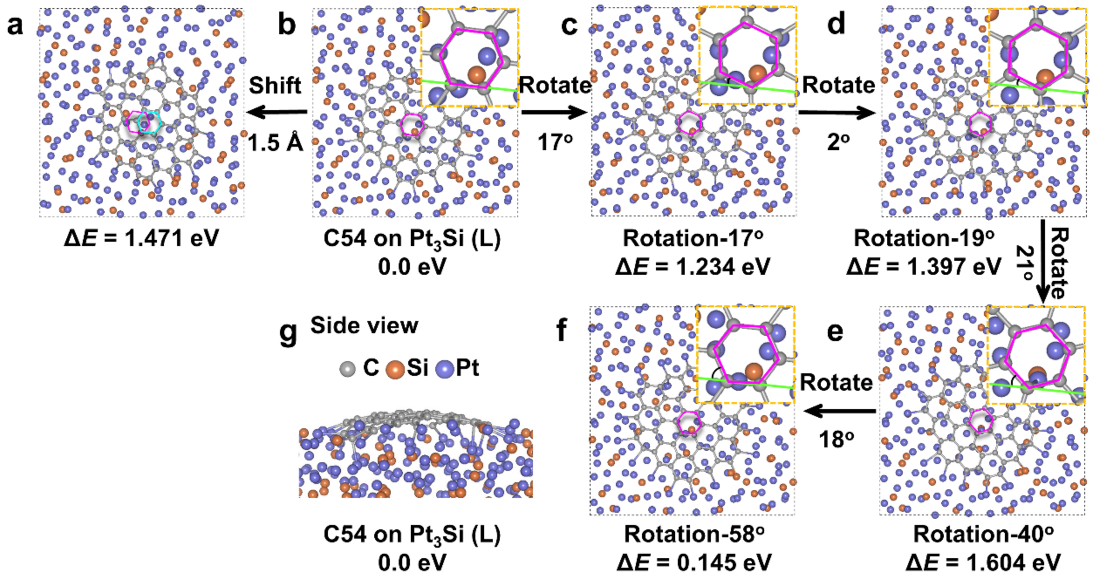

Supplementary Figure 15. The schematic diagrams of the atomic structures of graphene nucleus (C54) with different orientations and locations on Pt_3Si (L) surface. a, The schematic diagram of the atomic structure of C54 on Pt_3Si (L) surface after shifting 1.5 Å from (**b**). **b**, The schematic diagram of the initial atomic structure of C54 without movement on Pt_3Si (L) surface. **c-f**, The schematic diagrams of the atomic structures of C54 on Pt_3Si (L) surface after rotating 17° (**c**), 19° (**d**), 40° (**e**) and 58° (**f**) from (**b**). **g**, The side-view schematic diagram of the atomic structure of C54 on Pt_3Si (L) surface. The relative energies of all structures were calculated taking the structure with the lowest total energy as the reference, and shown below each corresponding schematic diagram.

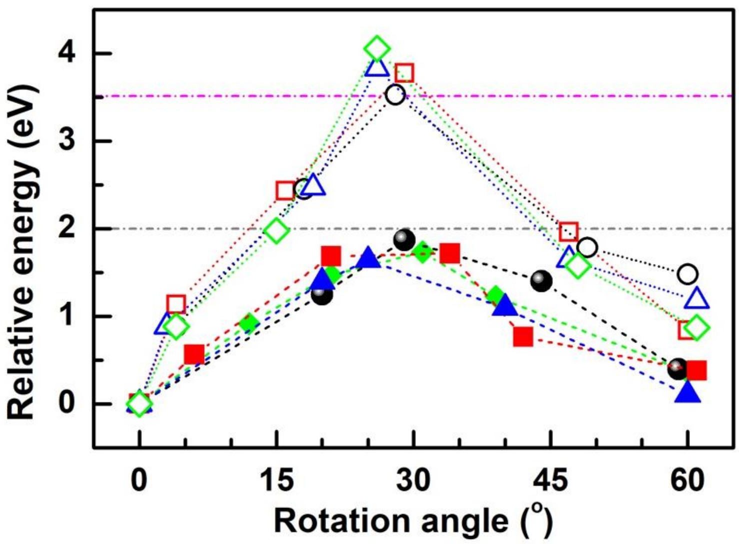

Supplementary Figure 16. The relative energies of graphene nucleus with different orientations on Pt (111) and Pt_3Si (L) surfaces. We considered the rotation of graphene nucleus by randomly select four structures from the last 740 ab initio MD simulation steps for both Gr/Pt (111) and Gr/Pt_3Si (L). The dotted lines with hollow symbols denote Gr/Pt (111), and the dash lines with solid symbols denote Gr/Pt_3Si (L). Note that the highest relative energies for graphene nucleus rotation on Pt (111) are larger than 3.5 eV, while those on Pt_3Si (L) are less than 2.0 eV for all four cases.

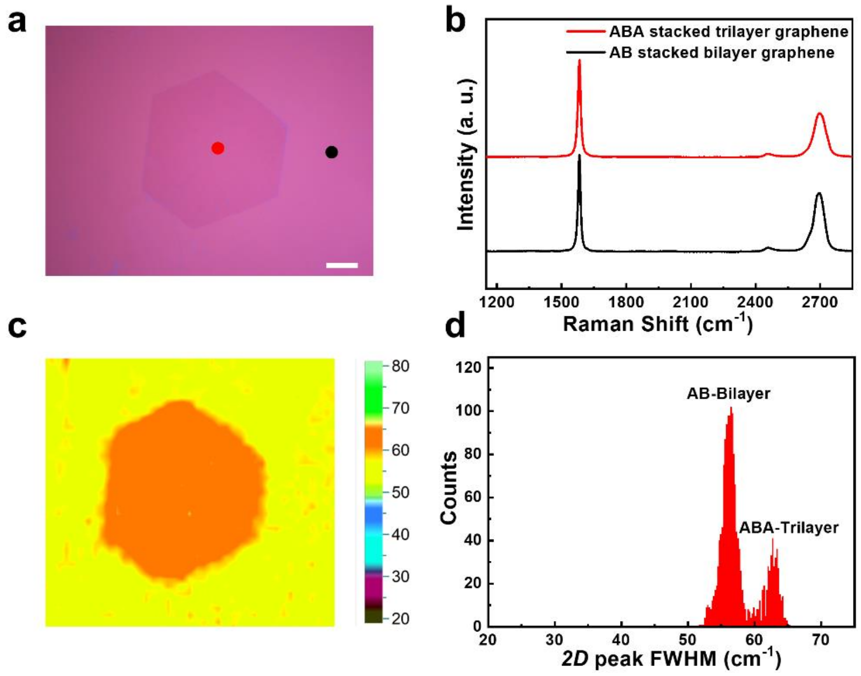

Supplementary Figure 17. Characterization of the trilayer graphene domains grown on the liquid Pt_3Si/solid Pt substrate by interlayer epitaxy. a, Optical image of a trilayer domain, in which the hexagonal third layer was grown on a continuous AB-BLG film. **b,** Raman spectra with normalized G peak intensity taken from the marked points in (**a**). **c,** *2D* peak FWHM Raman map of the trilayer graphene region in (**a**). **d,** Statistical histograms of the *2D* peak FWHM extracted from the Raman map in (**c**). Note that the *2D* peak FWHM of the trilayer domain is in the range of 60 – 65cm^{-1}, indicating ABA stacking[1]. Scale bar: **a**, 10 μm.

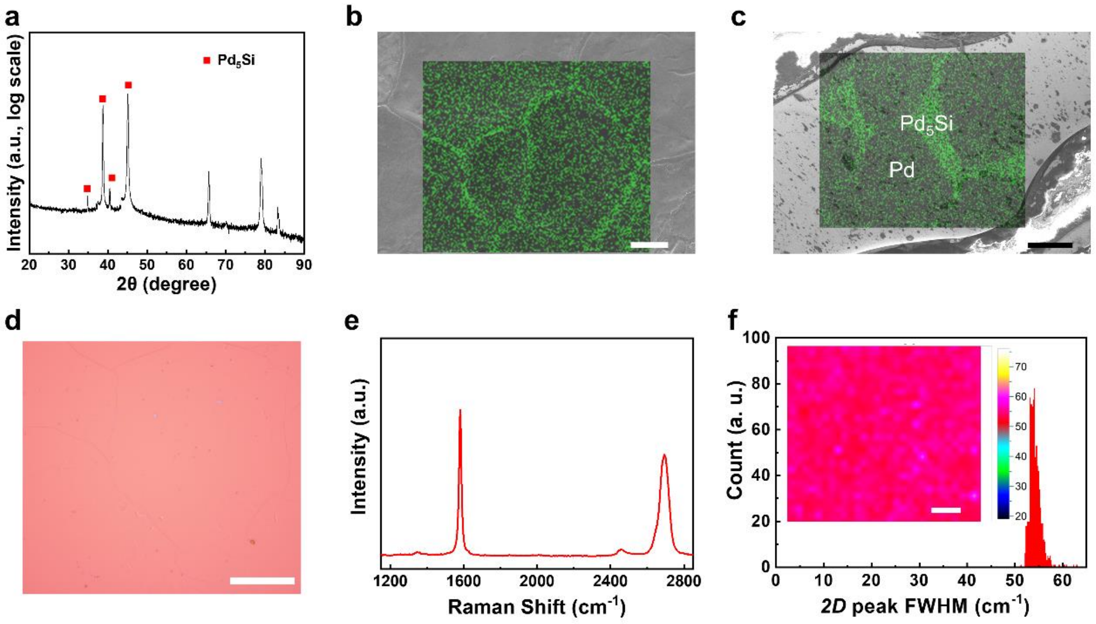

Supplementary Figure 18. Characterizations of Pd_5Si/Pd substrate and the AB-BLG film grown on it. a, XRD pattern of the substrate. **b,c,** EDS maps of the Si Kα1 peak overlapped with the corresponding SEM images of the surface (**b**) and cross section (**c**) of the substrate. Note that the Si element is distributed on the surface and the grain boundaries of the Pd foil, forming a shell-core structure of Pd_5Si/Pd. **d,** Optical image of the AB-BLG film transferred onto SiO_2/Si substrate. **e,** A typical Raman spectrum of the AB-BLG film in (**d**). **f,** Raman map and the corresponding statistical histogram of *2D* peak FWHM of the AB-BLG film in (**d**), showing that the *2D* peak FWHM is in the range of 52 – 58 cm^{-1}, typical feature of AB stacking. Raman mapping area is 150 × 150 μm^2 with a spot size of 1 μm and step of 1 μm. Scale bars: **b,c**, 100 μm; **d,f**, 50 nm.

Supplementary References

1. Lui, C. H. *et al.* Imaging stacking order in few-layer graphene. *Nano Lett.* **11**, 164-169 (2011).
